# Toward Trial Readiness in Congenital Myotonic Dystrophy: A Longitudinal Cohort Study of Predictors of Motor Function in Childhood

**DOI:** 10.1212/NXG.0000000000200363

**Published:** 2026-04-01

**Authors:** Michael Kiefer, Julia M. Hartman, Kiera N. Berggren, Amanda Butler, Aileen S. Jones, Melissa McIntyre, Man Hung, Samuel Carrell, Melissa A. Hale, Craig Campbell, Valeria Ada Sansone, Nicholas E. Johnson

**Affiliations:** 1 Center for Inherited Myology Research, Virginia Commonwealth University, Richmond;; 2 Department of Physical Therapy, Virginia Commonwealth University, Richmond;; 3 Department of Neurology, Virginia Commonwealth University, Richmond;; 4 Department of Pediatric Neurology, University of Utah, Salt Lake City;; 5 Roseman University of Health Sciences, Salt Lake City, UT;; 6 Department of Pediatrics, London Children’s Hospital, University of Western Ontario, Canada; and; 7 The NeMO Clinical Center in Milan, Neurorehabilitation Unit, University of Milan, Italy.

## Abstract

**Background and Objectives:**

Congenital myotonic dystrophy (CDM) is a life-limiting genetic disorder present at birth, marked by profound motor and cognitive impairments. CDM is the most severe form of myotonic dystrophy type 1 (DM1), both caused by a cytosine–thymine–guanine (CTG) repeat expansion in the DM1 protein kinase gene. Clinical trials targeting the shared disease mechanism in DM1 adults have shown promise. However, a lack of validated clinical end points in early childhood and a limited understanding of the variable disease progression are challenges to trial design in CDM. This longitudinal cohort study identifies predictors of motor function in children with CDM to inform future clinical trials.

**Methods:**

Children with genetically confirmed CDM participated in a longitudinal natural history study, completing annual motor assessments, including the 10-m walk/run (10MWRT), 6-minute walk test, supine to stand, and grip strength. Perinatal clinical features and early childhood motor milestone achievement were captured through caregiver proxy report. Linear mixed-effects models were used to evaluate predictors of concurrent motor performance and 1-year change. The relationship between [MBNL]_inferred_, a biomarker of splicing dysregulation, and the achievement of motor milestones was evaluated in a subset of participants who provided muscle biopsy samples.

**Results:**

A total of 138 visits from 42 children with CDM aged 3.0–15.3 years were included in the analysis. Motor performance on all motor outcomes improved with age. In age-adjusted models, age at independent walking explained more variance in 10MWRT times (*R*^2^ = 0.31, β = 0.2, *p* = 0.003) than CTG repeats_per100_ (*R*^2^ = 0.25, β = 0.2, *p* = 0.018). Baseline motor function was the strongest predictor of 1-year change across all outcomes, with lower baseline performance associated with greater improvement. More severe splicing dysregulation during adolescence was associated with later age at independent walking (rho = −0.708, *p* = 0.001).

**Discussion:**

The severity of clinical course in infancy and age of independent walking are key predictors of motor function across childhood in CDM. However, baseline motor performance is the strongest predictor of 1-year change and should be a central consideration in clinical trial design. Larger, prospective studies are needed to assess the responsiveness of early motor milestones as indicators of treatment response.

## Introduction

Myotonic dystrophy type 1 (DM1) is an autosomal dominant disorder caused by a CTG repeat expansion in the DM1 protein kinase (DMPK) gene.^1-4^ This life-limiting neuromuscular disorder results in motor, cognitive, and cardiac impairments.^1,5^ The most severe phenotype, congenital myotonic dystrophy (CDM), is associated with large intergenerational expansions of CTG repeat size and results in severe and highly variable symptom progression throughout childhood.^1^ The wide range in age of symptom onset and severity in DM1 is linked to, but not fully explained by CTG repeat size.^5,6^ Currently, disease-modifying clinical trials have been conducted in adults with DM1, targeting the shared disease mechanism. However, clinical trials in children with CDM are hindered by a limited understanding of factors contributing to the highly variable disease progression, as well as the lack of validated clinical end points for early childhood.

Symptoms of CDM typically present during the perinatal period and include severe hypotonia, respiratory problems, feeding difficulties, and clubfoot.^7,8^ There is a 30% mortality rate in the first year of life for children requiring ventilatory support for 3 months or more.^9^ Children who survive the initial period of critical illness often show functional improvement during childhood, followed by variable decline in adolescence and adulthood.^10,11^ Despite early gains, children with CDM continue to exhibit motor and cognitive impairments compared with age-matched neurotypical peers. In adolescence, progressive motor, cognitive, and cardiac impairments emerge, resembling the adult-onset presentation. Ultimately, fewer than 50% of individuals with CDM survive into their mid-30s.^12^ Natural history studies have identified several motor outcome measures that are both feasible and reliable in children older than 3 years.^10,11^ Yet, limited data exist regarding the factors contributing to performance variability across childhood.

The primary aim of this study is to identify patient characteristics associated with variability in motor outcome measure performance throughout childhood and adolescence. A secondary aim is to examine the relationship between disease severity in infancy and the clinical course throughout childhood to identify potential targets for clinical trial end points in young children with CDM who have not yet achieved independent ambulation.

## Methods

### Study Design

Enrollment in the first study, health endpoints and longitudinal progression in congenital myotonic dystrophy (HELP-CDM) (n = 52), began in 2014, and the second study, trial readiness and endpoint assessment in congenital myotonic dystrophy (TREAT-CDM) (n = 57, NCT03059264), began enrollment in 2017. Participants who completed HELP-CDM were eligible to enroll in TREAT-CDM, resulting in a total of 80 unique participants in the combined studies. Study visits occurred every 12 months across both studies, yielding up to 6 observations per participant (3 per study) over a 60-month observation period (baseline, 12-month, and 24-month visits per study).

Enrollment criteria for both studies required a clinical diagnosis of CDM with genetic confirmation of DM1 in the child or parent. Clinical diagnosis was operationally defined by the presence of myotonic dystrophy symptoms within the neonatal period (under 30 days), such as hypotonia, respiratory distress, feeding difficulties, or talipes equinovarus, necessitating hospitalization for more than 72 hours. Diagnosis of DM1 was confirmed through identification of an expanded CTG trinucleotide repeat in the DMPK gene in the child or an affected mother, with a repeat size exceeding 200 in the child considered diagnostic for DM1. Participants were excluded if they had other medical conditions that, in the opinion of site investigators, could affect motor performance. Only children 3 years and older completed functional testing; however, the collection of biospecimens and the medical intake form were completed for all enrolled participants.

### Procedures

The following functional assessments were performed using previously described methods^10,11^: 10-m walk/run test (10MWRT), 6-minute walk test (6MWT), supine to stand (STS), and grip strength (Grip). Grip was measured in kilograms force (kg) using the Jamar Plus + digital hand dynamometer (Sammons Preston, Warrenville, IL). Three trials of grip were conducted on both hands, and the average value across all valid trials was used in the analysis to minimize the effects of potential changes in reported hand dominance in this pediatric cohort over time. Within-subject changes in functional performance were calculated for 1-year intervals (305–425 days).

The size of the CTG repeat expansion was recorded from clinical records at the baseline. Medical and developmental histories were collected from caregiver reports at their first recorded visit across each study. These included the age at which the child began walking (age walking, in months), and we developed a CDM Severity Score based on caregiver report of the infant's initial presentation. The CDM Severity Score ranges from 0 to 5, with 1 point assigned for each of the following: decreased or absent fetal movements, ventilator support, neonatal intensive care unit (NICU) admission longer than 7 days, feeding problems, and unilateral or bilateral clubfoot. Items for this exploratory scale were selected based on their clinical relevance and data availability. The following questions from the study's Medical Intake form, filled out by the primary caregiver, were used to determine scores presented as radio buttons “item: discrete response options.” Response options that increased the CDM Severity Score are underlined (each equals 1 point).

1. Fetal movement: normal, decreased, absent.
2. Any of the following interventions for breathing problems at birth? Oxygen by nose or mask, nasal continuous positive airway pressury (CPAP), intubation/ventilation (tube down to lungs/on ventilator), none.
3. History of NICU stay? Yes, No.

If yes, how long was the NICU stay? ≤ 48 hours, >48 hours and <1 week, ≥1 week.

1. Feeding problems? Yes, No.
2. Club Foot: none, left, right, bilateral (both feet).

Age walking was also determined by caregiver report on the Medical History Intake Form through the following prompt: “Age participant began to walk? ___________”

### Statistical Approach

Linear mixed-effects models were used to examine predictors of motor function at the time of visit (concurrent models) and predictors of 1-year change in motor function. Additional models, concurrent adjusted for age and 1-year change adjusted for baseline function, were fit to determine how these relationships vary across age and functional levels. Velocities were calculated for the 10MWRT (10 m/time = meters/s) and the STS (1STS/time = 1/s) to align with recent analysis approaches^13^ in other neuromuscular conditions and account for baseline functional differences. Percent of predicted values for age-matched and sex-matched unaffected peers was used for 6MWT^14^ and grip^15^ to account for age-related and sex-related differences across childhood. Owing to the limited age range (5–17) of predicted values for the 6MWT,^14^ 11 observations from 8 participants younger than 5 years were excluded from analysis. All values for the grip were retained as reference values^15^ for ages 3–17 years were available.

Analyses were performed in R version 4.5.1 (2025-06-13). Only participants with complete predictor data were included in statistical models to allow for model comparisons. Models were estimated using maximum likelihood through the lmer function from the lme4 package.^16^ A *p* value of less than 0.05 was considered statistically significant. Linear random-intercept models examined the association between key predictors (sex, CTG repeats, Severity Score, and age walking) and performance on the 10MWRT, 6WMT, STS, and grip. Marginal *R*^2^ was calculated using the Nakagawa approach^17^ to assess the variance explained by fixed effects. Descriptive statistics for longitudinal measures (e.g., age at visit, motor function test performance) were derived from all available visit data. For characteristics collected only at baseline (e.g., CTG repeat length, age walking, Severity Score), values were taken from each participant's earliest recorded entry. Cross-study subject linkage was used to prevent duplicate inclusion of individuals' fixed characteristics.

### Correlation of MBNL_[inferred]_ and Achievement of Walking

A biomarker of alternative splicing dysregulation, muscleblind-like [MBNL]_inferred_, reflective of the molecular pathology of DM1, was measured in tissue samples of the vastus lateralis in a subset of participants (n = 17) using previously described methods.^18-20^ [MBNL]_inferred_ ranges from 0 to 1, where 1 reflects full MBNL activity as in a healthy muscle and 0 represents complete loss of MBNL activity, indicating severe dysfunction. Previous studies show that [MBNL]_inferred_ can vary over time within individuals with CDM, with an overall trend toward improvement in early childhood, followed by increasing variability beginning around age 7 years.^18,20^ To examine the relationship between early childhood motor performance and variability of splicing dysregulation in adolescence, analyses involving [MBNL]_inferred_ were completed in participants 6 years and older. The relationship between [MBNL]_inferred_ and age walking was evaluated using Spearman correlation^21^ and an independent samples *t* test.

### Standard Protocol Approvals, Registrations, and Patient Consents

Children with CDM, aged 0–15 years, were enrolled in 2 sequential longitudinal observational cohort studies conducted at Virginia Commonwealth University, the University of Utah, the University of Western Ontario in Canada, and the NEuroMuscular Omnicentre (NEMO) in Milan, Italy. All study procedures received approval from the Institutional Review Boards at each participating site before enrollment. Written informed consent was obtained from 1 parent, and verbal assent was obtained from children aged 8 years and older as appropriate. Funding for US sites was obtained from the NIH (Grant No. 1K23NS091511‐01 to NEJ). Funding in Italy was through Telethon (grant no. GUP19002 to VAS). Funding in Canada was through the Children's Health Foundation.

### Data Availability

Anonymized data not published within this article will be made available by request from any qualified investigator.

## Results

### Participant Characteristics

Of 80 subjects and a total of 303 visits completed across both studies, 42 children (138 visits) had complete predictor data and were included in the primary analysis (Table 1). Most exclusions were due to missing CTG repeat values, which were available for 52 subjects. Among the 42 participants included, the majority, 31 children (74%), had CTG repeat lengths ranging from 500 to 1,500. Across the full cohort, CTG repeat lengths ranged from 400 to 2,530. The median reported age walking was 24 months (range: 11–48 months). The median severity score was 3 (range: 0–5). The most frequently reported severity score symptom was feeding problems 37/42 (88%), followed by NICU admission 34/42 (81%), while the least commonly reported symptom was unilateral or bilateral clubfeet 13/42 (31%). Seventeen participants between 6 and 16 years had [MBNL]_inferred_ values, which ranged from 0.275 to 0.933. The majority of the cohort performed well below age-matched peers, with markedly reduced percent-predicted grip strength (median 35.0 [IQR 25.3–43.6]) and 6MWT performance (median 58.5 [47.4–73.1]).

**Table 1:** Participant Characteristics

	Median (Q1–Q3)	Range	Participants/observations
Age at visit (y)	8.0 (5.8–10.6)	3.0–15.3	42/138
10MWRT (s)	5.5 (4.3–7.3)	1.8–18.4	38/113
10MWRT (meters/s)	1.8 (1.4–2.3)	0.5–5.5	38/113
6MWT (meters)	325 (250–417)	87–604	38/117
6MWT (percent of predicted)	58.5 (47.4–73.1)	16.3–103.0	38/106
STS (s)	5.4 (3.3–10.0)	1.8–23.3	37/112
STS (1/s)	0.2 (0.1–0.3)	0.0–0.6	37/112
Grip (kilograms)	4.5 (2.9–6.0)	0.6–22.8	39/121
Grip (percent of predicted)	35.0 (25.3–43.6)	4.7–134.8	39/121
Age walking (mo)	24 (18–27)	11–48	42 participants
Severity score	3 (2–4)	0–5	42 participants
(MBNL)inferred	0.73 (0.58–0.83)	0.28–0.93	17 participants
Age walking: Before 18 mo = 10 (24%), 18 mo or later = 32 (76%)			
Sex: male = 24 (57%), female = 18 (43%)			

CTG repeats, (%)				
0–500 3 (7)	>500–1,000 16 (38)	>1,000–1,500 15 (36)	>1,500–2000 6 (14)	>2000 2 (5)

Clinical Features of Severity, (%)				
Fetal movement 25 (60)	Ventilator 15 (36)	NICU admit 34 (81)	Feeding problems 37 (88)	Clubfoot 13 (31)

Observations per participant, (%)					
1 visit 9 (21)	2 visits 7 (17)	3 visits 5 (12)	4 visits 8 (19)	5 visits 10 (24)	6 visits 3 (7)

Abbreviations: 10MWRT = 10-m walk/run test; 6MWT = Six-minute walk test; STS = supine to stand.

### Predictors of Motor Function at the Time of Visit

**Figure 1:**
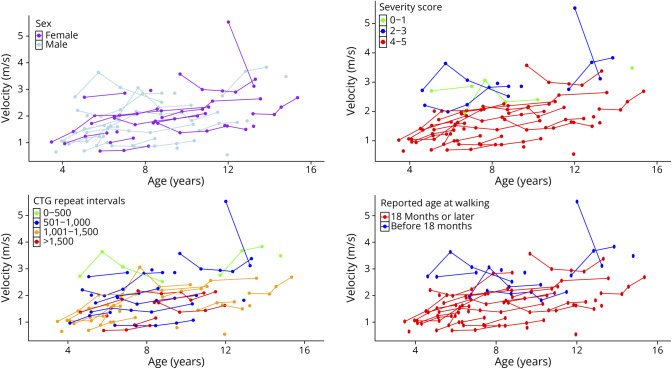
10MWRT Velocity by Age and Key Predictors Faster velocities were associated with lower clinical severity in childhood (lower severity score and earlier walking) and fewer CTG repeats. There was no relationship between 10MWRT velocity and sex. 10MWRT = 10-m walk/run test.

**Figure 2:**
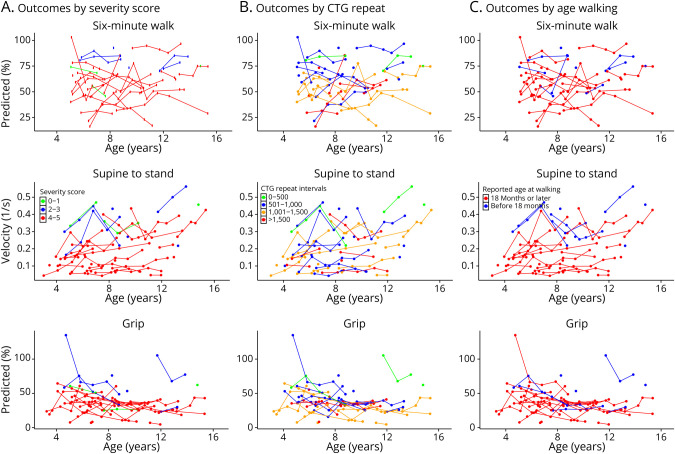
Panels Show Performance on the 6-Minute Walk (Percent of Predicted), Supine to Stand Velocity (1/s), and Grip Strength (Percent of Predicted) Tests (A) Color-coded by Severity Score (0–5; lower scores indicate fewer symptoms in infancy), showing better performance with lower severity. (B) Color-coded by CTG repeat bands, showing associations between shorter repeat length and better performance. (C) Color-coded by reported age of independent walking, with earlier walking associated with better performance.

We first sought to examine factors associated with variability in the overall disease course and to identify clinical end point targets in early childhood (e.g., age walking) that predict future functional outcomes. To do this, we fit models to predict concurrent (same-visit) motor outcomes data based on sex, age at visit, CTG repeat length, and early life metrics of disease severity: age walking and severity score. Model results for concurrent (same-visit) motor outcomes are presented in the following tables: 10MWRT (Table 2), 6MWT (eTable 1A), STS (eTable 2A), and grip (eTable 3A). Visualizations between age and the predictors of interest are presented in Figure 1 for the 10MWRT and Figure 2 for the 6MWT, STS, and grip.

**Table 2:** Predictors of 10-m Walk/Run Test (Velocity, m/second)

Predictor	Unadjusted			Adjusted for age		
	Estimate (95% CI)	*p* Value	*R* ^2^	Estimate (95% CI)	*p* Value	*R* ^2^
Sex (male)	−0.23 (−0.78 to 0.32)	0.401	0.02	−0.11 (−0.60 to 0.39)	0.662	0.15
Severity score (0–5)	−0.42 (−0.60 to −0.25)	<0.001	0.31	−0.39 (−0.54 to −0.24)	<0.001	0.43
CTG repeats (per 100 repeats)	−0.10 (−0.15 to −0.05)	<0.001	0.25	−0.09 (−0.14 to −0.05)	<0.001	0.37
Age walking (mo)	−0.06 (−0.09 to −0.04)	<0.001	0.30	−0.05 (−0.07 to −0.03)	<0.001	0.38
Age (y)	0.11 (0.07–0.15)	<0.001	0.14			

One hundred thirteen observations from 38 subjects.

In unadjusted models, severity score, CTG repeat size, and age walking were significant predictors of most motor outcomes, while sex was not significant for any outcome. Age walking explained the most variance for STS (*R*^2^ = 0.26, β = −0.01, *p* < 0.001) and six-minute walk test (SMWT) (*R*^2^ = 0.20, β = −1.1, *p* < 0.001) and was similar to severity score for 10MWRT (*R*^2^ = 0.30, β = −0.06, *p* < 0.001 vs *R*^2^ = 0.31, β = −0.42, *p* < 0.001). Predictive models for grip explained lower proportions of variance. Severity score (β = −5.95, *p* = 0.005, *R*^2^ = 0.12) and CTG repeat size (β = −1.47, *p* = 0.012, *R*^2^ = 0.11) were the only significant predictors of grip.

In age-adjusted models, severity score, age walking, and CTG repeat size consistently predicted motor performance, while sex was not a significant predictor for any outcome. For the 10MWRT, severity score explained the largest proportion of variance (*R*^2^ = 0.43, β = −0.39, *p* < 0.001), followed by age walking (*R*^2^ = 0.38, β = −0.05, *p* < 0.001) and CTG repeats (*R*^2^ = 0.37, β = −0.09, *p* < 0.001). For STS velocity, the predictors performed similarly, with severity score (*r*^2^ = 0.33, β = −0.05, *p* < 0.001), age walking (*R*^2^ = 0.31, β = −0.01, *p* < 0.001), and CTG repeats (*R*^2^ = 0.30, β = −0.01, *p* < 0.001) all with similar explained proportions of variance. In the 6MWT, age walking (*R*^2^ = 0.20, β = −1.07, *p* < 0.001) and CTG repeats (*R*^2^ = 0.19, β = −1.77, *p* = 0.002) explained the most variance, with severity score contributing less (*R*^2^ = 0.08, β = −4.64, *p* = 0.030). For grip, severity score and CTG repeats explained similar variance (*R*^2^ = 0.15, β = −6.27, *p* = 0.004; β = −1.54, *p* = 0.009), slightly higher than age walking (*R*^2^ = 0.08, β = −0.65, *p* = 0.053), which was just below the a priori cutoff for statistical significance.

### Predictors of 1-Year Change in Motor Outcomes

**Figure 3:**
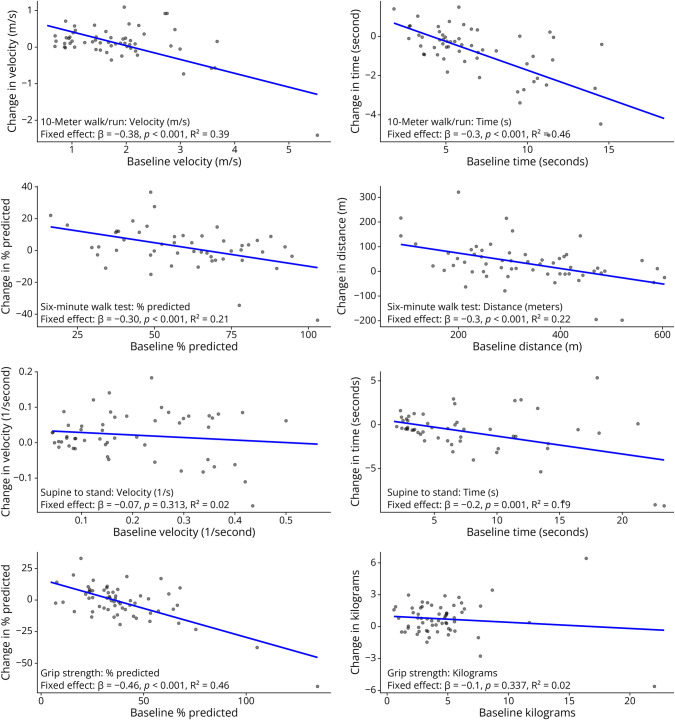
Worse Baseline Function Is Associated With Larger Improvements at 1 Year However, the strength of the association depends on the units used to calculate change.

To identify factors relevant to consider across shorter observation intervals, consistent with many clinical trial designs, we fit models of change in 10MWRT (Table 3), 6MWT (eTable 1B), STS (eTable 2B), and grip (eTable 3B). Comparison of baseline function as a predictor of motor outcomes at 1 year, using either raw scores or adjusted units (velocity for 10MWRT and STS and percent predicted for SMWT and Grip), is presented in Figure 3.

**Table 3:** Predictors of Change at 1 Year: 10-m Walk/Run Test (Velocity, m/second)

Predictor	Unadjusted			Adjusted for baseline function		
	Estimate (95% CI)	*p* Value	*R* ^2^	Estimate (95% CI)	*p* Value	*R* ^2^
Sex (male)	0.17 (−0.08 to 0.42)	0.177	0.03	0.16 (−0.13 to 0.45)	0.262	0.41
Severity score (0–5)	0.07 (−0.06 to 0.20)	0.271	0.02	−0.09 (−0.27 to 0.08)	0.264	0.42
CTG repeats (per 100 repeats)	0.01 (−0.02 to 0.04)	0.703	0.00	−0.03 (−0.07 to 0.00)	0.077	0.44
Age walking (mo)	0.01 (−0.01 to 0.03)	0.288	0.02	−0.03 (−0.05 to −0.00)	0.019	0.45
Age (y)	−0.03 (−0.07 to 0.01)	0.188	0.03	0.05 (−0.01 to 0.10)	0.096	0.45
Baseline (m/s)	−0.38 (−0.52 to −0.24)	<0.001	0.39			

Fifty seven observations from 26 subjects.

Baseline function was the only statistically significant predictor of 1-year change in motor function in unadjusted models. Baseline function explained the most variance in 10MWRT velocity (*R*^2^ = 0.39, β = −0.38, *p* < 0.001), 6MWT distance (*R*^2^ = 0.21, β = −0.30, *p* < 0.001), and grip strength (*R*^2^ = 0.46, β = −0.46, *p* < 0.001). By contrast, STS velocity showed no significant association with baseline function (*R*^2^ = 0.02, β = −0.07, *p* = 0.313) or any other predictor, including Age walking, age, sex, severity score, or CTG repeat length.

After adjusting for baseline performance, there were no statistically significant associations between percent change at 1 year and additional predictors, with a few exceptions for age walking and the ambulator outcomes. After adjusting for baseline performance, including age walking led to improvements in explained variance in 1-year change in 10MWRT velocity (*R*^2^ = 0.45, β = −0.03, *p* = 0.019) and 6MWT distance (*R*^2^ = 0.40, β = −0.68, *p* = 0.024).

Notably, the strength of the association between baseline function and 1-year outcomes varied depending on whether change was computed in raw scores or units such as velocity or percent predicted (Figure 3). This was most evident in STS, where there was a statistically significant relationship and a larger proportion of variance explained using time in seconds (*R*^2^ = 0.19, β = −0.2, *p* = 0.001) compared with velocity, which had no statistically significant relationship (*R*^2^ = 0.02, β = −0.07, *p* = 0.313). However, the reverse was true in models for grip, where the association between baseline function and 1-year change was only observed when change was modeled in percent of predicted values, (*R*^2^ = 0.46, β = −0.46, *p* < 0.001) compared with unadjusted kilograms (*R*^2^ = 0.02, β = −0.1, *p* = 0.337). It is important to note that although 2 models failed to demonstrate statistical significance, the overall direction of the relationship remained consistent, with worse baseline function linked to greater improvements at 1 year.

### Early Life Motor Function Correlates With [MBNL]inferred Biomarker in Adolescence

**Figure 4:**
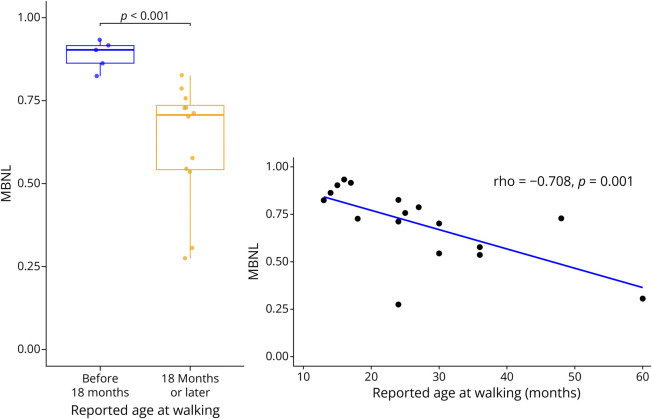
The Welch t Test for Comparison of Groups With Unequal Variance Mean MBNL in children who walked before 18 months of age was higher (0.89) compared with children who walked at 18 months or later (0.62), *p* < 0.001. Higher MBNL values in adolescence were correlated with earlier age of independent walking (rho = −0.708, *p* = 0.001).

Our data thus far suggest that the early life predictor age walking performed the best at predicting our functional motor outcomes in childhood. To evaluate the relationship between age walking and [MBNL]_inferred_, a biomarker reflective of the primary disease process, we tested its association in adolescent children with CDM (Figure 4). [MBNL]_inferred_ is a composite measure of RNA splicing dysregulation in DM1, where lower values correlate with more severe disease. We found that higher [MBNL]_inferred_ values were correlated with earlier age walking (rho = −0.708, *p* = 0.001). When grouping subjects based on walking achievement before or after 18 months, the World Health Organization (WHO) upper bound of norm-referenced values for independent walking,^22^ we found children who walked before 18 months had significantly better splicing regulation (mean [MBNL]inferred = 0.89) compared with those who walked at 18 months or later (mean [MBNL]inferred = 0.62), *p* < 0.001.

## Discussion

Recent advancements in therapeutic interventions for adults with DM1 highlight the urgent need to prepare for clinical trials in children with CDM, the most severe form of DM1. This study examined predictors of motor function in a large, longitudinal cohort of children with CDM. These data provide some potential tools to guide clinicians in long-term prognosis. Specifically, disease severity, as measured by reported age of achieving independent walking (age walking) and severity of symptoms in infancy (severity score), is associated with overall motor performance throughout childhood. Findings from this study may inform clinical trial design, including eligibility criteria and end point selection. When modeling 1-year change in motor outcomes, baseline motor function emerged as a stronger predictor than age, CTG repeat length, or early-life severity markers. This suggests that while early developmental milestones may inform the overall prognosis, function at baseline is the strongest predictor of change during a shorter time interval (e.g., 1 year). In addition, [MBNL]_inferred_, a measure of disease-associated splicing dysregulation in DM1 skeletal muscle measured during adolescence, was strongly correlated with the age of independent walking, supporting the idea that early motor milestones reflect the underlying disease burden. This finding suggests that delays in early motor development may serve as a functional indicator of disease-associated molecular pathology in adolescence and that early intervention using therapeutics that improve global splicing dysregulation has the potential to modify the phenotypic trajectory of CDM children. Overall trends of improving motor function from infancy through early childhood, previously reported from 1-year observations in this cohort,^10,11^ were also seen here using extended follow-up and an alternative analytic approach. However, in age-normalized measures for the 6MWT and grip, lack of association between age and percent predicted suggests that strength and endurance gains do not outpace those of typically developing peers.

We found that early childhood markers of disease severity, severity score, and age of independent walking predicted performance across multiple motor outcomes, even after adjusting for age. While severity score attempts to capture a broader representation of clinical presentation in infancy and still has implications for predicting future motor function, the timing of many of these components (i.e., fetal movements/ventilatory support) may limit the ability to prospectively collect these data or target them as an end point in clinical trials. Therefore, clinical trials may target age of walking achievement or other early motor milestones as surrogate end points, a strategy that has shown success in clinical trials for infants with spinal muscular atrophy (SMA).^23-25^ Clinician-reported assessments, such as the WHO Motor Milestones (WHO-MM) scale,^22^ may offer more reliable operational milestone definitions than caregiver report, which can be subject to recall bias or inconsistent interpretation of “independence”. Trucco et al.^26^ report a higher prevalence of delays in early milestones (head control, sitting, walking) in children with CDM compared with children with childhood-onset DM1, a milder phenotype, providing additional evidence that early milestone achievement is reflective of the overall severity of the disease course. Earlier milestones, such as head control and sitting, may be preferred for earlier detection of treatment effects, as some children in this cohort did not achieve independent walking until as late as 5 years of age.

A second key finding is that baseline motor performance was the strongest predictor of 1-year functional change, even after accounting for age. This finding aligns with previous studies in children with Duchenne muscular dystrophy, which also showed that baseline function is a critical predictor of change.^27-29^ Factors that predict short-term, 1-year, or 2-year change in motor function may be especially important to consider in clinical trial design. For example, a recent Phase III therapeutic trial of tideglusib (AMO pharma, NCT03692312) in children with CDM older than 6 years failed to meet its primary clinical end point, the Clinician-Completed CDM Type 1 Rating Scale (a disease-specific clinician-rated scale). Accounting for baseline function and variability in disease progression could improve the sensitivity of future trials to detect treatment-related benefits or harms. In addition, variation in the strength of the association between baseline function and 1-year change is dependent on whether change is calculated as raw scores or velocities/percent of predicted. This highlights the importance of considering the most appropriate units to quantify change given the baseline function and age range of the sample. Further studies of cohorts that match clinical trial inclusion criteria and stratify analyses by age will be important to inform clinical trial design.

Theoretically, earlier therapeutic intervention is optimal for progressive conditions such as DM1. However, children younger than 6 years have been excluded from clinical trials, presumably due to the lack of clinical endpoints for this population. Complicating matters, children with CDM demonstrate functional improvements in early childhood even without therapeutic intervention, making it difficult to detect treatment effects. Norm-referenced developmental assessments that account for age-referenced motor gains result in floor effects and feasibility challenges in this population.^10^ In addition, outcome measures such as the 10MWRT, 6MWT, and strength, although feasible and responsive in older children, are not suitable for infants or nonambulatory toddlers. Infant scales such as the Children's Hospital of Philadelphia Infant Test of Neuromuscular Disorders^30^ and the Hammersmith Infant Neurologic Examination Motor Milestones (HINE-2), which primarily assess early motor skills such as head control, rolling, and up to walking in the HINE-2,^31^ have been used as endpoints in pivotal trials^23,25^ of spinal muscular atrophy,^32^ which also presents with severe weakness in infancy. However, children with CDM show spontaneous improvement after the perinatal period of critical illness, and in our cohort, many achieve independent walking by age 2 years (Table 1). Often, clinical trials last 52 weeks or longer, and children may not enroll immediately after birth due to diagnosis delays or medical acuity. Therefore, many children would likely reach ceiling effects in these scales during the course of a clinical trial. Scales that are sensitive across a wide range of skills, such as the Gross Motor Function Measure,^33^ which assesses abilities from lying and rolling through running and jumping, may be more appropriate for adaptation and use in CDM. While originally designed to measure motor function in children 5 months to 16 years with cerebral palsy, there is emerging evidence for its use in other populations with cognitive and motor impairments.^34^

It is important to consider several limitations of this study. Most importantly, the age of independent walking and CDM severity score were based on caregiver report survey responses, which introduces the potential for recall bias and subjective interpretation of the prompts. Second, although this is one of the largest cohorts of children with CDM studied to date, the overall sample size remains small, and some models relied on data from as few as 25 participants and 54 visits. In addition, a small subset of participants contributed disproportionately to the data set, with half of the participants (n = 21) contributing 100 of the 138 total visits. Furthermore, the strength of some of these associations appears dependent on the units used to represent the outcome measure (seconds vs velocities, percent predicted vs kilograms, etc.). Replication of these findings in larger, independent cohorts with more balanced participant representation will be important to confirm the generalizability of these results and further investigate how these relationships vary across ages. Finally, although previous research supports the reliability of motor assessments in this population,^10^ cognitive impairments may still contribute to variability in test performance and bias the sample towards less severe phenotypes that can complete testing. The items included in the severity scale were chosen based on their clinical relevance and the availability of parent-reported data. Further research is needed to determine which aspects of infantile severity are most clinically meaningful and best predict future functional outcomes.

In conclusion, this study demonstrates that early childhood markers of disease severity, such as the age of independent walking and severity score is a strong predictor of future motor performance in children with CDM. This finding may be clinically valuable when evaluating young children for trial enrollment or therapeutic intervention. Moreover, 1-year changes in motor performance seem to be linked to baseline function, emphasizing the importance of considering baseline performance in clinical trial design.

## Glossary

10MWRT: 10-m walk/run test
6MWT: 6-minute walk test
CDM: congenital myotonic dystrophy
CPAP: continuous positive airway pressure
CTG: cytosine thymine–guanine
DM1: myotonic dystrophy type 1
DMPK: DM1 protein kinase
HELP-CDM: health endpoints and longitudinal progression in congenital myotonic dystrophy
HINE-2: hammersmith infant neurologic examination motor milestones
MBNL: muscleblind-like
NICU: neonatal intensive care unit
SMA: spinal muscular atrophy
SMWT: six-minute walk test
STS: supine to stand
TREAT-CDM: trial readiness and endpoint assessment in congenital myotonic dystrophy
WHO: World Health Organization
